# The association between sleep quality and anxiety symptoms: a cross-sectional study based on Tibetan university students at high altitude in China

**DOI:** 10.3389/fpsyg.2025.1505948

**Published:** 2025-03-28

**Authors:** Mengying Shi, Ruijing Miao, Meijun Bing, Shiru Liu

**Affiliations:** ^1^School of Marxism, Xinjiang Normal University, Urumqi, China; ^2^Information Management Center, Xinjiang Normal University, Urumqi, China; ^3^Xinjiang University Library, Urumqi, China; ^4^Department of Physical Education and Research, Xinjiang University, Urumqi, China

**Keywords:** high altitude, correlations, lifestyle, sleep quality, mental health

## Abstract

**Background:**

There have been more previous studies on sleep quality and anxiety symptoms among university students in plains areas. However, fewer studies have been conducted on Tibetan university students at high altitude. Analyzing the relationship between sleep quality and anxiety symptoms due to increased altitude may provide a reference for mental health promotion and intervention for Tibetan university students in high altitude areas.

**Methods:**

Stratified whole cluster sampling was used. The pittsburgh sleep quality index (PSQI) scale was used to investigate the sleep quality of 4,777 university students at high altitude in China. The self-rating anxiety scale (SAS) was used to investigate anxiety symptoms. The chi-square test was used to compare the detection rates of anxiety symptoms among different groups, and logistic regression analysis was used to analyze the association between sleep quality and anxiety symptoms.

**Results:**

The prevalence of anxiety symptoms among Tibetan university students at high altitude in China was 25.7%. The detection rate of anxiety symptoms among girls (28.4%) was higher than that of boys (22.1%), and the difference was statistically significant (*χ*^2^ = 24.634, *p* < 0.001). The detection rates of anxiety symptoms among university students with sleep quality of “Good,” “Medium” and “Poor” were 1.8, 4.8 and 39.2%, respectively, and the differences were statistically significant (*χ*^2^ = 779.759, *p* < 0.001). Logistic regression analysis after adjusting for relevant covariates showed that the risk of anxiety symptoms was higher in the “Medium” (OR:3.479, 95%CI:1.946 ~ 6.221) and “Poor” (OR:44.817, 95%CI:29.175 ~ 68.847) groups, compared with the “Good” group. The same trend was observed in both men and women at the university level.

**Conclusion:**

There is a close relationship between sleep quality and the occurrence of anxiety symptoms among Tibetan university students at high altitude in China. Improving sleep quality may have a positive effect on reducing the occurrence of anxiety symptoms among Tibetan university students at high altitude.

## 1 Introduction

With the increasing pressure of life and employment, coupled with the constant changes in the natural environment and lifestyle, the occurrence of anxiety symptoms among university students is prevalent and shows an ever-increasing trend (Peres et al., 2017). A World Health Organization World Mental Health Survey of 18–22 year olds in 21 countries showed that 20.3% of the population suffered from anxiety and only 16.4% sought treatment for mental disorders (Auerbach et al., 2016). A meta-analysis that included 100,187 university students in 64 studies showed that the prevalence of anxiety symptoms among university students was 39% (95% CI, 34.6–43.4%). The prevalence of anxiety symptoms was highest in North America (48.3, 95% Cl 37.4–59.2%), low- and middle-income countries (54.2, 95% Cl 35.0–73.4%), and the United States of America (Li W. et al., 2022). A survey of a large public university in the United States showed that 86% of university students had disrupted sleep patterns, and another 71% had increased stress and anxiety due to COVID-19 outbreaks (Son et al., 2020). A survey of Malaysian university students aged 18–24 showed that 34% of Malaysian university students had moderate anxiety problems and 29% had severe or very severe anxiety problems, and that the occurrence of anxiety problems was related to academic stress, gender (Shamsuddin et al., 2013). A study of Turkish university students also showed that 51% of the participants had moderate anxiety and there was a strong association with smoking behavior (Ayran et al., 2022). A survey of Chinese university students showed that moderate anxiety accounted for 2.7% and mild anxiety accounted for 21.3%, and that there was a correlation with urban and rural areas, family income, and parental status (Cao et al., 2020). It can be seen that the prevalence of anxiety symptoms is high among university students around the world and that there are many factors affecting anxiety symptoms (Hoge et al., 2017). University students are in the unstable period of psychological state, anxiety symptoms occur if not timely mitigation and intervention, will be very easy to lead to more serious mental health problems, and even produce suicidal behavior (Subar et al., 2022). It has also been shown that the development of anxiety symptoms is associated with a variety of factors such as lifestyle, sleep quality, dietary behaviors, physical activity (Crawford et al., 2019).

Sleep problems are spreading all over the world, affecting people’s lives and studies. At the same time, the occurrence of sleep quality problems is moving from the upper age group to the lower age group (Gusman et al., 2021). University students are also at an important stage in the development of sleep problems, which deserves attention and concern. A survey of university students in 26 high-income and middle-income countries showed that 39.2 per cent of university students slept for less than 6 h (Peltzer and Pengpid, 2016). Additionally, there are studies confirming that 55–60% of university students report poor sleep quality (Lund et al., 2010). A survey of 7,626 university students aged 18–29 in the United States also showed that 43% of the university students said that their sleep time > 30 min, and in the event of exams, increased stress when sleep quality is worse (Becker et al., 2018). China is no exception, with a survey of 3,423 Chinese university students showing that 24.42% had daytime sleep dysfunction (Zhang et al., 2024). Another PSQI survey of 1,227 university students in China also showed that 74.33% of the respondents had poor sleep quality and there was an association with depressive symptoms (Chen et al., 2022). Research has shown that the development of sleep quality problems can lead to a range of adverse effects or problems, including decreased cognitive function, increased risk of chronic disease, decreased fitness levels, decreased academic performance, and increased risk of chronic disease (Gardani et al., 2022; Suardiaz-Muro et al., 2020). At the same time, the occurrence of sleep quality will also lead to the occurrence of various kinds of bad moods, resulting in various kinds of psychological problems or diseases (Xiong et al., 2021).

There is a growing body of research on the relationship between sleep quality and various psychological problems among university students (Baldini et al., 2025; Kentiba et al., 2018; Tanaka-Matsumi and Kameoka, 1986). A meta-analysis showed a weighted effect size of 0.39 between sleep quality and stress among university students, and a slightly higher effect size of 0.41 between insomnia and stress (Gardani et al., 2022). A survey of Nigerian undergraduates also showed that 50.1%(95%CI:45.7–54.5%) had poor sleep quality, and the presence of psychological distress, anxiety symptoms, was also significantly associated with poor sleep quality (Seun-Fadipe and Mosaku, 2017). A survey of 12,922 university students in China showed that sleep quality was associated with interpersonal sensitivity, anxiety, and depressive symptoms, and did not change according to gender (Xu et al., 2022). It can be seen that there are more studies on university students’ sleep quality and depression and psychological stress, and relatively few studies on anxiety symptoms. Other studies have shown that the sleep quality of university students at high altitude is a worrying problem and should be given sufficient attention and concern (Pramsohler et al., 2019). A survey of university students from two universities in Tibet, China, confirmed that the prevalence of poor sleep quality, anxiety symptoms, and depressive symptoms were 45.69, 36.81, and 51.86%, respectively, which were higher than those in the plains, and that there was a significant and direct effect between poor sleep and anxiety/depression severity (Wang Y. et al., 2023). A study on college students in Erzurum, Türkiye, 1800 meters above sea level, shows that there is a close relationship between sleep quality and anxiety symptoms of college students (Uygur et al., 2024).

Past studies have shown that there are more studies on sleep and mental health for university students, including studies on sleep quality and psychological symptoms, depression and anxiety, and the results are not consistent. However, unfortunately, past studies have mainly focused on university students in plain areas, and there are fewer studies on the relationship between sleep quality and anxiety symptoms among university students in high altitude areas. As a typical high-altitude area in the world, Tibetans in China have lived in this area for a long time, forming a unique plateau adaptation (Peng et al., 2022a,b; Zhou et al., 2022). It is not clear whether there is an association between sleep quality and anxiety symptoms in this group. Therefore, this study investigated 4,777 Tibetan university students at high altitude to analyze the association between sleep quality and anxiety symptoms. The aim is to provide the necessary reference for mental health promotion and intervention for Tibetan university students at high altitude. The hypothesis of this study is that there is a negative correlation between sleep quality and anxiety symptoms of Tibetan university students in high-altitude areas, that is, the better the sleep quality, the lower the anxiety symptoms.

## 2 Methods

### 2.1 Participants

**Figure 1:**
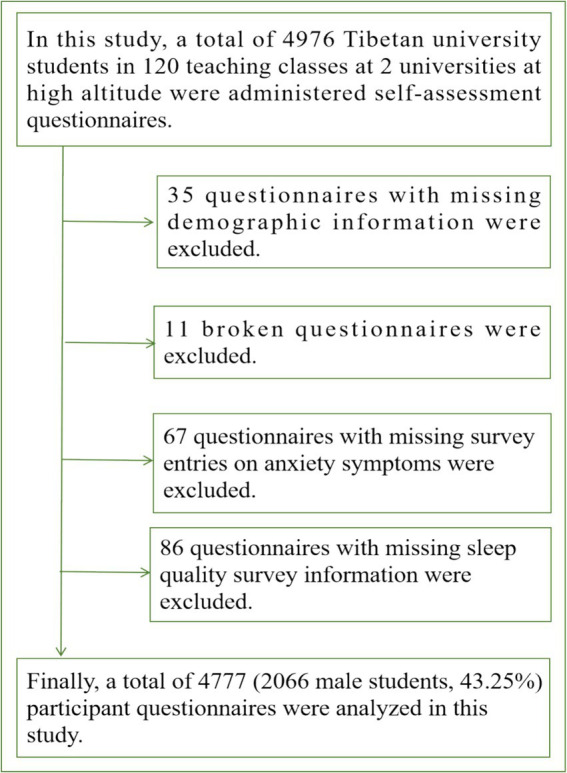
Sampling and exclusion process of Tibetan university students in high altitude areas of China.

In this study, whole population sampling was used to sample the participants. In Lhasa (The altitude is 3,650 meters), Tibet, China, 2 universities were selected as test schools for the participants of this study. Classes with more than 80% of Tibetan undergraduates in the university’s classes were used as sampled classes. The classes were coded and each grade level was coded as 1,2,3,4,…; then 15 teaching classes were randomly selected for each grade in the order of first year of university to fourth year of university. The inclusion conditions of this study were: both father and mother were Tibetan; they had been living and studying at high altitude in Tibet since birth; they were 19–22 years old; Under the evaluation of the teaching staff, it is believed that they do not have any significant psychological or physiological illnesses; and they voluntarily accepted the investigation of this study and gave informed consent. A total of 4,976 Tibetan university students from 120 teaching classes in 2 universities were surveyed in this study. After excluding 199 invalid questionnaires after the survey, a total of 4,777 valid questionnaires were returned (2,066 male students, 43.25%). In this study, a self-assessment questionnaire on sleep quality and anxiety symptoms was administered to 4,777 Tibetan university students at high altitude in China. Among them, 2066 (43.25%) were male students with a mean age of (20.30 ± 1.07) years. There were 2,711 female students (56.75%) with a mean age of (20.05 ± 0.99) years. This study was approved by the Ethics Committee of Xinjiang Normal University (202387675). The specific sampling process is shown in Figure 1.

### 2.2 Measures

#### 2.2.1 Basic demographic information

The basic demographic information of the participants in this study included information such as year of birth, gender, region, school, class, ethnicity, father’s and mother’s ethnicity, and the date of the survey. The age of the participants was calculated based on their year of birth and date of survey. Participants’ ages were rounded to the nearest whole number, e.g., 18 years means 18.0–18.9 years.

#### 2.2.2 Anxiety symptoms

In this study, the self-rating anxiety scale (SAS) was used to investigate the current situation of anxiety symptoms (Dunstan and Scott, 2020). This scale was developed by Zung (1971). and is a self-reported scale that effectively assesses participants’ anxiety symptoms. The scale consists of 20 items reflecting anxiety symptoms, such as “I feel scared for no reason,” “I feel like I’m going to collapse,” “I feel like my heart is beating fast,” etc. Each item is rated from 1 to 4 on a scale from “none or very little” to “most or all of the time.” Each entry was rated on a scale of 1–4 from “none or very little” to “most of the time or all of the time.” Participants made a single choice for each entry based on their actual situation in the past 7 days. The 20 entries were categorized into negative and positive experiences, with the positive experience entries (5,9,13,17,19) being reverse scored. The 20 entry scores were summed to obtain the raw score for the scale, which ranged from 20 to 80 points. The raw score was multiplied by 1.25 to obtain the standardized score of the scale. The standardized score is 100 points. The <50 cut-off was defined as no anxiety symptoms, the 50–59 cut-off as mild anxiety symptoms, the 60–69 cut-off as moderate anxiety symptoms, and the >69 cut-off as severe anxiety symptoms. In this study, the participants were categorized into two groups: those with no anxiety symptoms (<50 points) and those with anxiety symptoms (≥50 points), and the SAS was found to be effective in measuring participants’ anxiety symptoms, with a Cronbach’s alpha =0.83 (Tanaka-Matsumi and Kameoka, 1986).

#### 2.2.3 Sleep quality

The pittsburgh sleep quality index (PSQI) scale was used in this study for the assessment of participants’ sleep quality. The PSQI was developed in a study by Buysse et al. (1989). The scale is a self-assessment scale used to assess participants’ sleep quality over the past 30 days. The scale yields 7 factor scores and an overall score reflecting sleep quality. The 7 factors are: subjective sleep quality, sleep latency, sleep duration, sleep efficiency, sleep disturbance, use of hypnotic medication, and daytime dysfunction. Scores for each factor ranged from 0 to 3, and the total PSQI score was 0–21. Higher participant scores indicate poorer sleep quality. In this study, participants’ sleep quality was categorized as Good (PSQI ≤5), Medium (PSQI 6–7), and Poor (PSQI >7) (Windmill et al., 2024).

#### 2.2.4 Covariates

The covariates investigated in this study include age, father’s education, mother’s education, breakfast frequency, screen time, body mass index (BMI), physical activities. Age was calculated based on the time of testing and date of birth, rounded to the nearest whole number. The division of father’s education and mother’s education is divided into: elementary school and below, middle school, college and above. Breakfast frequency of the main survey participants consumed breakfast in the past 7 days. This study was categorized as ≤1 time/week, 2–4 time/week, and ≥ 5 time/week. The classification of screen time was based on the participants’ average screen time per day in the past 7 days. Based on the average screen time per day, they were categorized as: ≤2 h/d, >2 h/d (Madigan et al., 2019). BMI is calculated based on the results of height and weight tests. BMI=Weight (Kg)/Height (m)^2^. Based on the results of BMI, they are categorized as underweight (18.4Kg/m^2^), normal (18.5–23.9Kg/m^2^), overweight (24.0–27.9Kg/m^2^), obese (≥28Kg/m^2^) (Hu et al., 2017). The physical activities survey was based on the physical activities section of CNSSCH Association (2022). Participants were surveyed for the past 7 days, including 5 weekdays and 2 weekends. Participants were asked to recall the frequency and duration of their physical activities in the past 7 days according to their own circumstances, so as to calculate the average duration of their physical activities in the past 7 days. In this study, only the time of moderate-to-vigorous physical activities (MVPA) was calculated, which was categorized as <30 min/d, 30–60 min/d, and > 60 min/d according to relevant studies.

#### 2.2.5 Quality control

In this study, questionnaires were administered using participant self-assessment. The questionnaires were administered by trained teachers. The survey was divided into two groups who entered the school classes at the same time to administer the questionnaire. Each group consisted of about 2–3 staff members. The main purpose of the study survey and the requirements of the survey were explained to the participants before the questionnaire survey. After the questionnaires were distributed, students were asked to fill them out independently without interference from others. Questionnaire filling time was about 15–25 min. The questionnaires were handed over to the staff promptly after completion and checked for completion. The tests of height and weight involved in this study were conducted by specialized personnel. The testing method was in accordance with the method required by CNSSCH Association (2022). The height was accurate to 0.1 cm and the weight was accurate to 0.1 kg.

### 2.3 Statistical analysis

Before data analysis, strict cleaning of the data is carried out, including the removal of extreme values, completion of missing values, and testing of data normality, to ensure the accuracy of data analysis and results. The prevalence of anxiety symptoms among Tibetan university students at high altitude in China was expressed as a percentage. Comparison of the detection rates of anxiety symptoms among different covariates was performed by chi-square test. Logistic regression analysis was used to analyze the relationship between sleep quality and anxiety symptoms. In the logistic regression analysis, the presence of anxiety symptoms among Tibetan university students was used as the dependent variable, and sleep quality was used as the independent variable. Crude Model did not control covariates. Model1 controlled age, father’s education, and mother’s education. Model2 further controlled breakfast frequency, screen time, BMI, physical activities on the basis of Model1. Data were processed and analyzed using SPSS 25.0 software.

## 3 Results

Overall, the prevalence of anxiety symptoms among Tibetan university students at high altitude in China was 25.7% (1,226/4777). The detection rate of anxiety symptoms was 22.1% (456/2066) for boys and 28.4% (770/2711) for girls, and the difference was statistically significant (*χ*^2^ value was 24.634, *p* < 0.001). The proportions of Tibetan university students with sleep quality of “Good,” “Medium” and “Poor” in high-altitude areas of China were 26.1, 10.9 and 10.9%, and the differences were statistically significant (*χ*^2^ value was 38.892, *p* < 0.001).

The detection rate of anxiety symptoms among Tibetan university students in high altitude areas of China was statistically significant when compared with different father’s education, mother’s education, breakfast frequency, and physical activities (*χ*^2^ value 21,881, 24,115, 43,170, 13,565, *p* < 0.001). In terms of sleep quality, the detection rate of anxiety symptoms among Tibetan university students with sleep quality of “Good” was 1.8%, “Medium” was 4.8% and “Poor” was 39.2%, and the difference was statistically significant (*χ*^2^ value 779.759, *p* < 0.001). Comparison of the detection rates of anxiety symptoms by gender is shown in Table 1.

**Table 1:** Comparison of the detection rate of anxiety symptoms among Tibetan university students in high altitude areas of China (%).

Variables	Boys (*n* = 2066)				Girls (*n* = 2,711)				Total (*n* = 4,777)			
	N	%	*χ*^2^-value	*P*-value	N	%	*χ*^2^-value	*P*-value	N	%	*χ*^2^-value	*P*-value
Age (years)												
19	126	21.1	6.193	0.103	285	28.9	2.981	0.395	411	25.9	2.916	0.405
20	128	20.6			235	26.7			363	24.2		
21	104	21.5			175	30.7			279	26.5		
22	98	26.9			75	27.4			173	27.1		
Father’s education												
Elementary school and below	167	26.9	23.231	<0.001	224	31.0	5.624	0.060	391	29.1	21.881	<0.001
Middle school	273	21.3			491	28.0			764	25.2		
College and above	16	9.8			55	23.2			71	17.8		
Mother’s education												
Elementary school and below	239	24.2	8.856	0.012	359	29.5	17.503	<0.001	598	27.2	24.115	<0.001
Middle school	203	20.9			395	28.9			598	25.6		
College and above	14	12.8			16	12.3			30	12.6		
Breakfast frequency												
≤1 time/week	118	34.9	57.026	<0.001	74	33.5	12.513	0.002	192	34.3	43.170	<0.001
2–4 time/week	161	25.1			205	32.7			366	28.9		
≥5 time/week	177	16.3			491	26.3			668	22.6		
Screen time												
≤2 h/d	86	14.9	23.908	<0.001	212	31.9	5.373	0.023	298	24	2.398	0.131
>2 h/d	370	24.8			558	27.3			928	26.2		
**BMI**												
Underweight	52	23.3	1.214	0.750	167	29.6	7.079	0.069	219	27.8	5.397	0.145
Normal	241	22.6			388	26.9			629	25.1		
Overweight	86	21.8			57	25.6			143	23.1		
Obese	77	20.2			158	32.6			235	27.1		
Physical activities (MVPA)												
< 30 min/d	331	23.6	7.852	0.020	663	29	8.267	0.016	994	26.9	13.965	0.001
30–60 min/d	102	20.0			89	23.5			191	21.5		
> 60 min/d	23	14.9			18	40.9			41	20.7		
Sleep quality (PSQI)												
Good	10	1.6	313.485	<0.001	13	2.1	457.064	<0.001	23	1.8	779.759	<0.001
Medium	15	6.8			10	3.3			25	4.8		
Poor	431	35.6			747	41.6			1,178	39.2		
Total	456	22.1			770	28.4			1,226	25.7		

BMI, Body mass index; MVPA, Moderate-to-vigorous physical activities; PSQI, Pittsburgh sleep quality index.

Table 2 shows the logistic regression analyses of different covariates and anxiety symptoms among Tibetan university students at high altitude in China. Overall, the detection rates of anxiety symptoms were lower among fathers (OR: 0.525, 95% CI: 0.396–0.697) and mothers (OR: 0.385, 95% CI: 0.260–0.571) with “College and above” education compared to the “Elementary school and below” group (*p* < 0.001).Compared with the group with breakfast frequency of “≥5 time/week” as a reference, Tibetan university students with breakfast frequency of “2–4 time/week” (OR: 1.788, 95% CI: 1.472–2.172) and “≤1 time/week” (OR: 1.388, 95% CI: 1.196–1.611) had a higher risk of anxiety symptoms (*p* < 0.001). The results of the analysis by sex are shown in Table 2.

**Table 2:** Logistic regression analysis of different covariates and anxiety symptoms among Tibetan university students at high altitude in China.

Variables	Boys			Girls			Total		
	OR	95% CI	*P*-value	OR	95% CI	*P*-value	OR	95% CI	*P*-value
Age (years)									
19	1.000			1.000			1.000		
20	0.969	0.735 ~ 1.277	0.821	0.897	0.732 ~ 1.100	0.296	0.910	0.773 ~ 1.071	0.255
21	1.026	0.765 ~ 1.375	0.865	1.091	0.871 ~ 1.367	0.447	1.029	0.862 ~ 1.228	0.754
22	1.377	1.016 ~ 1.866	0.039	0.928	0.688 ~ 1.252	0.626	1.062	0.863 ~ 1.307	0.571
Father’s education									
Elementary school and below	1.000			1.000			1.000		
Middle school	0.733	0.587 ~ 0.916	0.006	0.868	0.719 ~ 1.049	0.142	0.819	0.710 ~ 0.946	0.007
College and above	0.295	0.171 ~ 0.510	<0.001	0.673	0.479 ~ 0.946	0.023	0.525	0.396 ~ 0.697	<0.001
Mother’s education									
Elementary school and below	1.00			1.00			1.000		
Middle school	0.826	0.668 ~ 1.022	0.078	0.972	0.820 ~ 1.152	0.744	0.923	0.809 ~ 1.053	0.234
College and above	0.461	0.258 ~ 0.822	0.009	0.335	0.196 ~ 0.574	<0.001	0.385	0.260 ~ 0.571	<0.001
Breakfast frequency									
≥5 time/week	1.000			1.000			1.000		
2–4 time/week	2.758	2.093 ~ 3.633	<0.001	1.408	1.045 ~ 1.896	0.024	1.788	1.472 ~ 2.172	<0.001
≤1 time/week	1.724	1.356 ~ 2.193	<0.001	1.362	1.119 ~ 1.657	0.002	1.388	1.196 ~ 1.611	<0.001
Screen time									
≤2 h/d	1.000			1.000			1.000		
>2 h/d	0.799	0.661 ~ 0.966	0.021	1.888	1.459 ~ 2.442	<0.001	1.126	0.969 ~ 1.309	0.122
BMI									
Underweight	1.000			1.000			1.000		
Normal	0.961	0.682 ~ 1.352	0.818	0.877	0.707 ~ 1.087	0.231	0.869	0.726 ~ 1.041	0.127
Overweight	0.915	0.619 ~ 1.354	0.658	0.816	0.575 ~ 1.160	0.257	0.781	0.612 ~ 0.996	0.046
Obese	0.830	0.557 ~ 1.237	0.36	1.152	0.886 ~ 1.498	0.29	0.966	0.778 ~ 1.199	0.753
Physical activities (MVPA)									
> 60 min/d	1.000			1.000			1.000		
30–60 min/d	1.762	1.112 ~ 2.791	0.016	0.589	0.321 ~ 1.082	0.088	1.412	0.994 ~ 2.007	0.054
< 30 min/d	1.420	0.867 ~ 2.326	0.163	0.443	0.232 ~ 0.846	0.014	1.046	0.716 ~ 1.529	0.815
Sleep quality (PSQI)									
Good	1.000			1.000			1.000		
Medium	4.514	1.997 ~ 10.204	<0.001	1.594	0.691 ~ 3.678	0.274	2.676	1.505 ~ 4.759	0.001
Poor	34.425	18.23 ~ 65.008	<0.001	33.094	18.953 ~ 57.785	<0.001	34.369	22.606 ~ 52.254	<0.001

BMI, Body mass index; MVPA, Moderate-to-vigorous physical activities; PSQI, Pittsburgh sleep quality index.

The overall results showed that after adjusting for the relevant covariates, logistic regression analysis between the factors of sleep quality and anxiety symptoms among Tibetan university students in high altitude areas of China showed significant differences (*p* < 0.05) between the analyses in terms of subjective sleep quality, sleep latency, sleep duration, habitual sleep efficiency, sleep disturbance, use of sleep medication, and daytime dysfunction (*p* < 0.05). Subjective sleep quality was “Very bad,” sleep latency was “>60 min,” sleep duration was “<5 h,” habitual sleep efficiency is “<65%,” sleep disturbance is “10–18,” Use of Tibetan university students with sleep medication of “≥3 Time/Week” and daytime dysfunction of “Big problem” had a higher risk of developing anxiety symptoms (*p* < 0.001). The results of logistic regression analysis by gender are shown in Table 3.

**Table 3:** Logistic regression analysis of PSQI and anxiety symptoms among Tibetan university students in high altitude areas of China.

Variables	Boys			Girls			Total		
	Adjusted *OR*	95% CI	*P*-value	Adjusted OR	95% CI	*P*-value	Adjusted OR	95% CI	*P*-value
Subjective sleep quality									
Very good	1.00			1.00					
Fairly good	3.47	2.015 ~ 5.967	<0.001	3.24	2.204 ~ 4.748	<0.001	3.22	2.359 ~ 4.387	<0.001
Fairly bad	18.27	10.537 ~ 31.665	<0.001	20.45	13.642 ~ 30.668	<0.001	18.56	13.474 ~ 25.577	<0.001
Very bad	27.64	15.968 ~ 47.838	<0.001	25.06	16.621 ~ 37.779	<0.001	24.56	17.785 ~ 33.927	<0.001
Sleep latency									
≤15 min	1.00			1.00					
16–30 min	1.42	0.998 ~ 2.020	0.051	1.26	0.962 ~ 1.648	0.094	1.33	1.077 ~ 1.647	0.008
31–60 min	4.43	3.159 ~ 6.222	<0.001	3.91	2.998 ~ 5.111	<0.001	4.07	3.309 ~ 5.005	<0.001
>60 min	8.91	6.338 ~ 12.531	<0.001	6.82	5.178 ~ 8.985	<0.001	7.50	6.069 ~ 9.272	<0.001
Sleep duration									
>7 h	1.00			1.00					
6–7 h	1.32	0.878 ~ 1.978	0.183	1.23	0.890 ~ 1.706	0.208	1.29	1.001 ~ 1.657	0.049
5–6 h	1.76	1.264 ~ 2.443	0.001	2.66	2.014 ~ 3.505	<0.001	2.23	1.805 ~ 2.748	<0.001
<5 h	2.43	1.77 ~ 3.338	<0.001	3.56	2.732 ~ 4.648	<0.001	3.04	2.488 ~ 3.725	<0.001
Habitual sleep efficiency									
≥85%	1.00			1.00					
75–84%	1.42	0.970 ~ 2.074	0.072	1.72	1.307 ~ 2.266	<0.001	1.61	1.293 ~ 2.009	<0.001
65–74%	2.42	1.710 ~ 3.435	<0.001	2.09	1.578 ~ 2.780	<0.001	2.15	1.732 ~ 2.679	<0.001
<65%	3.11	2.274 ~ 4.256	<0.001	3.98	3.093 ~ 5.108	<0.001	3.52	2.903 ~ 4.277	<0.001
Sleep disturbance									
0	1.00			1.00					
1–9	3.33	2.202 ~ 5.033	<0.001	0.97	0.724 ~ 1.312	0.864	1.60	1.266 ~ 2.028	<0.001
10–18	29.71	18.787 ~ 46.994	<0.001	11.06	7.989 ~ 15.319	<0.001	16.04	12.372 ~ 20.795	<0.001
19–27	13.54	8.810 ~ 20.812	<0.001	9.84	7.200 ~ 13.458	<0.001	11.02	8.618 ~ 14.088	<0.001
Use of sleep medication									
≤0 Time/Month	1.00			1.00					
<1 Time/Week	14.24	9.384 ~ 21.621	<0.001	43.72	30.131 ~ 63.444	<0.001	25.21	19.337 ~ 32.868	<0.001
1–2 Time/Week	16.24	11.048 ~ 23.881	<0.001	44.69	31.240 ~ 63.938	<0.001	26.40	20.532 ~ 33.937	<0.001
≥3 Time/Week	27.10	18.956 ~ 38.749	<0.001	35.41	25.477 ~ 49.218	<0.001	28.62	22.682 ~ 36.111	<0.001
Daytime dysfunction									
No problem	1.00			1.00					
Minor Problems	2.43	1.617 ~ 3.639	<0.001	1.94	1.309 ~ 2.888	0.001	2.23	1.687 ~ 2.958	<0.001
A few problems	4.51	3.029 ~ 6.703	<0.001	3.61	2.447 ~ 5.317	<0.001	4.08	3.103 ~ 5.370	<0.001
Big problem	7.74	5.120 ~ 11.704	<0.001	11.96	7.997 ~ 17.896	<0.001	10.51	7.915 ~ 13.965	<0.001

Logistic regression model controlled covariates such as age, father’s education, mother’s education, breakfast frequency, screen time, BMI and physical activities. OR, Odds Ratio; 95%CI, 95% Confidence Interval.

Stepwise logistic regression analyses were performed with anxiety symptoms as the dependent variable and sleep quality as the independent variable among Tibetan university students at high altitude in China. The results showed that in general, compared with the “Good” group, the risk of anxiety symptoms was higher in the “Medium”(OR: 3.479, 95%CI:1.946 ~ 6.221) and “Poor” groups (OR: 44.817, 95%CI:29.175 ~ 68.847) (*p* < 0.001). The results of the analysis of sleep quality and anxiety symptoms by sex are shown in Table 4.

**Table 4:** Logistic regression analysis of sleep quality and anxiety symptoms among Tibetan university students at high altitude in China.

Sex/Sleep quality (PSQI)	Crude model		Model 1		Model 2	
	OR	95%CI	OR	95%CI	OR	95%CI
Boys						
Good	1.000		1.000		1.000	
Medium	4.514	(1.997 ~ 10.204)^a^	4.591	(2.029 ~ 10.389)^a^	5.040	(2.216 ~ 11.465)^a^
Poor	34.425	(18.23 ~ 65.008)^a^	33.441	(17.702 ~ 63.176)^a^	33.905	(17.801 ~ 64.576)^a^
*P* for trend	<0.001		<0.001		<0.001	
Girls						
Good	1.000		1.000		1.000	
Medium	1.594	(0.691 ~ 3.678)	1.583	(0.686 ~ 3.653)	2.676	(1.146 ~ 6.248)
Poor	33.094	(18.953 ~ 57.785)^a^	32.737	(18.74 ~ 57.188)^a^	59.391	(33.203 ~ 106.235)^a^
*P* for trend	<0.001		<0.001		<0.001	
Total						
Good	1.000		1.000		1.000	
Medium	2.676	(1.505 ~ 4.759)	2.657	(1.493 ~ 4.726)	3.479	(1.946 ~ 6.221)^a^
Poor	34.369	(22.606 ~ 52.254)^a^	33.764	(22.203 ~ 51.345)^a^	44.817	(29.175 ~ 68.847)^a^
*P* for trend	<0.001		<0.001		<0.001	

Crude Model did not control for covariates. Model1 controlled for age, father’s education, mother’s education. Model2 further controlled for breakfast frequency, screen time, BMI, and physical activities on the basis of Model1. OR, Odds Ratio; 95% CI, 95% Confidence Interval.

a Indicates *p* < 0.001.

## 4 Discussion

The prevalence of anxiety symptoms among university students is increasing and affecting their lives, studies and mental health (Jin et al., 2022). The emergence of anxiety symptoms, if not intervened and guided in a timely manner, can lead to more serious mental health problems and even suicidal ideation, negatively affecting their quality of life (Li X. et al., 2022). However, there are many factors affecting the occurrence of anxiety symptoms in university students, which are influenced by a combination of lifestyle, dietary behaviors, sleep quality, geographic environment, gender, family environment, physical exercise, and many other factors (Berdida and Grande, 2023; Chen et al., 2023; Gilles et al., 2006). The Tibet region in China, as a typical high-altitude region in the world, has certain impacts on the sleep quality and mental health of university students. The results of this study showed that the detection rate of anxiety symptoms among Tibetan university students in Tibet, China was 25.7%. This result is higher than the results of surveys conducted in the plains of China (22.34%) (Yu et al., 2023) and abroad for university students in Saudi Arabia (11.1%) (Mohammed et al., 2021) and the UAE (22.3%) (Awadalla et al., 2020). The results of the present study also showed that anxiety symptoms were common among Tibetan university students in high-altitude areas of China, and that adequate attention should be paid to them. The results of this study also showed that the detection rate of anxiety symptoms was higher among girls (28.4%) than boys (22.1%), which is consistent with the findings of many studies (Jones et al., 2002; Mao and Chen, 2023). Possibly because, while girls are more inclined to close themselves off when they have a problem, boys can often vent their anxious situations through sports, socializing, and so on.

The results of this study also showed that the detection rate of anxiety symptoms differed in several covariates, including father’s education, mother’s education, breakfast frequency, and physical activities. Studies have confirmed that there are many factors affecting anxiety symptoms in university students, among which family environment, lifestyle, and dietary behaviors are closely related to the occurrence of anxiety symptoms (Herbert, 2022; Kundu et al., 2022). In addition, a survey of 18–30 year old university students in India confirmed that participants’ level of physical activity (moderate and high) was significantly and negatively associated with anxiety symptoms scores (OR, 0.16 and 0.96, *p* = 0.001) (Ghrouz et al., 2019). A survey of university students in Wuhan, China also showed that high PA university students were independently associated with a significantly lower risk of developing depression (OR, 0.67, 95%Cl: 0.44–0.89) after adjusting for potential confounders (Feng et al., 2014). Other studies have also shown that sleep quality has an impact on the mental health of university students (Perotta et al., 2021).

The results of this study showed that the proportion of Tibetan university students with sleep quality of “Good,” “Medium,” and “Poor” in high altitude areas of China were 26.1, 10.9, and 62.9%, respectively. It can be seen that most of the Tibetan university students have relatively poor sleep quality. Related research confirms that the lack of oxygen in high altitude areas leads to relatively low sleep quality in humans. Although Tibetans living in this area for a long time have formed a unique adaptation mechanism to high altitude. However, for university students at high altitude, the double pressure of academic and employment may be an important reason for poorer sleep quality. It has also been found that high altitude areas with low vegetation cover cause insufficient oxygen content in the air, which is also an important reason for poorer sleep quality (Wang R. et al., 2023).

The present study also showed that the detection rate of anxiety symptoms among Tibetan university students with sleep quality of “Good” was 1.8%, “Medium” was 4.8%, and “Poor” was 39.2% in high altitude areas of China. The detection rate of anxiety symptoms among university students with sleep quality of “Good” was 1.8%, “Medium” was 4.8%, and “Poor” was 39.2%. This result suggests that the detection rate of anxiety symptoms is higher among Tibetan university students with poorer sleep quality in high altitude areas of China. A study of university students showed that poor sleepers had a higher risk of mental health problems compared with good sleepers, and there was a strong correlation between the two (Al-Ajlouni et al., 2020). Another study of 4,531 first-year university students also confirmed a significant positive correlation between sleep quality and depressive symptoms, especially among students with lower levels of psychological resilience (Zhou et al., 2021). In addition, sleep quality is associated with the body’s hormone secretion, which is highly susceptible to mood changes and the onset of anxiety symptoms, as demonstrated by a study of university students that confirmed that insomnia was associated with elevated fasting insulin (*β* = 0.12, *p* = 0.04) and elevated triglyceride levels (*β* = 1.85, *p* < 0.001), and that Stress (*p* = 0.020) and anxiety (*p* = 0.013) from university students’ life events mediated the relationship between insomnia and hypertriglyceridemia (Hsu and Chang, 2022). The results of this study showed that the association between sleep quality and anxiety symptoms was not altered by the increase in altitude. Therefore, improving sleep quality may also be an effective means to reduce anxiety symptoms at high altitude. The high-altitude environment has a significant impact on the anxiety symptoms of university students, with sleep quality playing a key mediating role (Zhao et al., 2019). In high-altitude areas, oxygen is scarce and air pressure is low, which can easily lead to a decrease in sleep quality, such as an increase in sleep breathing disorders, which can further exacerbate anxiety because sleep is crucial for emotional regulation (Batista et al., 2021). In addition, the physiological discomfort and psychological pressure brought by the high-altitude environment itself can indirectly exacerbate anxiety by affecting sleep, and sleep deprivation is positively correlated with social anxiety (Wang et al., 2013). In addition, cultural and genetic factors also have a certain impact on the sleep and anxiety symptoms of Tibetan students in high-altitude areas. The lifestyle and dietary habits of Tibetan people, which are different from those of Han people in plain areas, can have a certain impact on their mental health (Peng et al., 2022a,b). In addition, there is a close correlation between genetic factors formed by long-term high-altitude living and sleep quality, and genetic factors may mediate the relationship between sleep quality and anxiety symptoms (Kious et al., 2019).

There are some strengths and limitations of this study. First, to the best of our knowledge, this is the first study on the association between sleep quality and anxiety symptoms among Tibetan university students at high altitude in China. This study may provide a reference for future mental health promotion among Tibetan university students in high altitude areas. Secondly, this study used the internationally recognized PSQI and SAS scales for self-assessment questionnaires of sleep quality and anxiety symptoms respectively, the results of which can be compared horizontally with the results of many studies at home and abroad, and have a positive effect on the promotion of the health development of Tibetan university students at high altitude areas in China. However, there are some limitations in this study. First, this study was a cross-sectional survey, which could only analyze the association between sleep quality and anxiety symptoms among Tibetan university students at high altitude, and could not understand the causal association. Second, this study was conducted in only 2 universities at high altitude, and the sample size was limited. In the future, the scope and sample size of the survey should be increased to better promote the health of Tibetan university students in high-altitude areas.

## 5 Conclusion

A significant association was found between sleep quality and anxiety symptoms among Tibetan university students at high altitude in China. Poor sleep quality may be an important factor in the occurrence of anxiety symptoms, and this association did not change according to gender. In the future, during the intervention of anxiety symptoms among Tibetan university students at high altitude, the implementers can pay attention to the improvement of their sleep quality, so as to better promote the mental health development of Tibetan university students at high altitude.

## Data Availability

The raw data supporting the conclusions of this article will be made available by the authors, without undue reservation.
